# Dexamethasone Inhibits White Adipose Tissue Browning

**DOI:** 10.3390/ijms25052714

**Published:** 2024-02-27

**Authors:** Alejandra Paula Giordano, Sabrina Eliana Gambaro, Ana Alzamendi, Alejandro Ezequiel Harnichar, María Amanda Rey, Luisina Ongaro, Eduardo Spinedi, María Guillermina Zubiría, Andrés Giovambattista

**Affiliations:** 1 Neuroendocrinology Laboratory, Multidisciplinary Institute of Cellular Biology (IMBICE, CICPBA-CONICET-UNLP), La Plata 1900, Argentina; giordanoalejandrap@gmail.com (A.P.G.); sabrigambaro@gmail.com (S.E.G.); aalzamendi@imbice.gov.ar (A.A.); aeharnichar@gmail.com (A.E.H.); arey@imbice.gov.ar (M.A.R.); gzubiria@imbice.gov.ar (M.G.Z.); 2 Biology Department, School of Exact Sciences, Universidad Nacional de La Plata, La Plata 1900, Argentina; 3 Department of Pharmacology and Therapeutics, McGill University, Montréal, QC H3A 0G4, Canada; luisina.ongarogambino@mcgill.ca; 4 CENEXA (UNLP-CONICET), La Plata Medical School-UNLP, Calles 60 y 120, La Plata 1900, Argentina; spinedi@cenexa.org

**Keywords:** thermogenesis, beige adipocytes, glucocorticoids

## Abstract

White adipose tissue (WAT) regulates energy balance through energy storage, adipokines secretion and the thermogenesis process. Beige adipocytes are responsible for WAT thermogenesis. They are generated by adipogenesis or transdifferentiation during cold or β3-adrenergic agonist stimulus through a process called browning. Browning has gained significant interest for to its preventive effect on obesity. Glucocorticoids (GCs) have several functions in WAT biology; however, their role in beige adipocyte generation and WAT browning is not fully understood. The aim of our study was to determine the effect of dexamethasone (DXM) on WAT thermogenesis. For this purpose, rats were treated with DXM at room temperature (RT) or cold conditions to determine different thermogenic markers. Furthermore, the effects of DXM on the adipogenic potential of beige precursors and on mature beige adipocytes were evaluated in vitro. Our results showed that DXM decreased UCP-1 mRNA and protein levels, mainly after cold exposure. In vitro studies showed that DXM decreased the expression of a beige precursor marker (*Ebf2*), affecting their ability to differentiate into beige adipocytes, and inhibited the thermogenic response of mature beige adipocytes (*Ucp-1*, *Dio2* and *Pgc1α* gene expressions and mitochondrial respiration). Overall, our data strongly suggest that DXM can inhibit the thermogenic program of both retroperitoneal and inguinal WAT depots, an effect that could be exerted, at least partially, by inhibiting de novo cell generation and the thermogenic response in beige adipocytes.

## 1. Introduction

Adipose tissue (AT) is anatomically comprised mostly of White AT (WAT) and Brown AT (BAT). Both participate in body energy balance, WAT mainly through lipids storage and adipokines secretion and BAT as a thermogenic organ that dissipates energy to produce heat and thus maintain body temperature [1]. In contrast to white adipocytes, brown adipocytes contain small lipid droplets and are rich in mitochondria, which produce heat by uncoupling protein 1 (UCP-1) [2]. In addition to brown and white adipocytes, beige adipocytes are another class of thermogenic cells also harboured within WAT. In non-stimulated conditions, beige adipocytes are white-like adipocytes, but, under cold or β3-Adrenergic Receptor (ARβ3) agonists treatment [3], they acquire characteristics of brown adipocytes through a process called browning, which includes high expression levels of UCP-1 [4] and increased mitochondrial content. This latter process is induced by proliferator-activated receptor gamma coactivator 1 alpha (PGC1-α), which plays a central role regulating both mitochondrial biogenesis and function [5] and also drives peroxisome proliferator-activated receptors (PPAR) activation [6]. Another transcriptional factor necessary for beige adipocyte program activation is the PR domain containing 16 (PRDM16) [7], which is a pivotal molecule necessary for browning and beige adipocytes maintenance.

Beige adipocytes arise in WAT depots by two mechanisms: de novo adipogenesis from progenitors and/or trans-differentiation of mature white adipocytes [8,9]. At present, it is known that beige and white adipocytes share a common precursor [10], however, the identity pattern of beige precursor cells is not yet fully understood. In this regard, Early B-cell factor 2 (EBF2) was identified as a transcription factor necessary for beige precursor cells [11] and is required for beige adipocyte development in mice [12]. While Ebf2 is an specific marker of the beige cell linage, platelet-derived growth factor receptor-a (PDGFRα) was proposed as a bipotential marker of adipocyte progenitors, capable of differentiating into white or beige adipocytes depending on the stimulus received [13]. Contrary to Ebf2 actions, the multi-C2H2 zinc finger transcriptional coregulator ZFP423 is responsible for maintaining the white adipocyte phenotype, repressing the expression of key thermogenic genes [14]. In fact, deletion of ZFP423 in mice increases the number of *Ucp1*-expressing adipocytes in WAT [14]. More recently, it has been described that ZFP423-Ebf2 protein interaction recruits corepressors that block PPARγ2 binding to thermogenic gene enhancers [15].

Glucocorticoids (GCs) are a class of corticosteroids whose levels increase mainly in stressful conditions, acting throughout almost the whole body [16,17]. They have an important immunomodulatory function and are powerful regulators of metabolic process such as gluconeogenesis and lipolysis [18]. Dexamethasone (DXM) is a synthetic glucocorticoid widely used in clinical settings to treat several diseases (inflammation, allergies, edema and skin and kidney disorders, among others) due to its potent anti-inflammatory actions. Unfortunately, DXM chronic therapies are limited due to their side effects, such as central obesity, dyslipidemia and insulin resistance [19]. DXM is also used as a pro-adipogenic agent to induce differentiation in preadipocytes cultures [20].

Regarding thermogenesis, very little is known about DXM’s effects on WAT browning. Most reports have described GCs effects in BAT thermogenesis, showing tissue remodelling from BAT to WAT-like phenotype in DXM treated mice, including increased lipid droplet content, decreased mitochondrial number and lower respiratory complexes levels [21], as well as decreased *Ucp-1* mRNA levels [22]. Additionally, DXM inhibits the norepinephrine stimulated *Ucp-1* mRNA expression in a dose-dependent manner in HIB-1B brown adipose cells [23]. However, mice lacking glucocorticoid receptor (GR) in BAT did not show differences in thermogenic markers, such as UCP-1 and Type II Deiodinase (DIO2), neither in BAT morphology, indicating that BAT-specific GR-knockdown did not affect mice cold tolerance [24].

In vitro studies have described that DXM could affect the expression of *Ucp-1* in beige adipocytes [25] and that DXM treatment in mice for 6 weeks reduced the expression of Prdm16 in WAT [26]. There is still limited information regarding the impact of DXM on the thermogenesis of beige adipocytes. Since WAT browning enhances energy expenditure, reducing body weight and improving insulin sensitization [27], understanding how GCs could affect this process is a matter of interest. For this reason, we studied, in vivo, the effect of DXM on WAT browning and, in vitro, the actions of DXM on beige adipocyte precursor cells (APCs) and in beige mature adipocytes. Our results indicate that DXM decreased both thermogenic genes expression in mature beige adipocytes and precursor markers expression in beige APCs, thus supporting the case that DXM inhibited WAT browning by affecting both cellular targets in both WAT depots studied.

## 2. Results

### 2.1. DXM Treatment Effects in Body Weight, Caloric Intake and Metabolic Parameters

**Figure 1:**
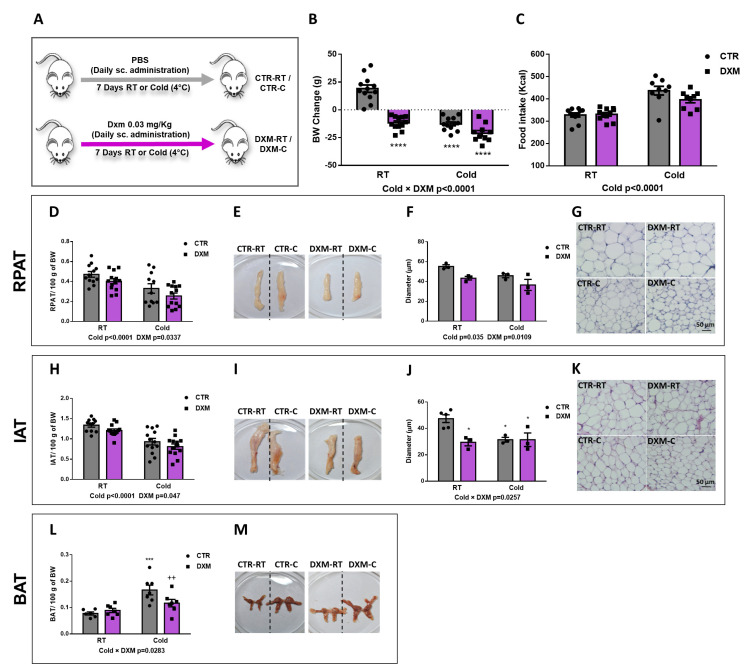
In vivo effects of DXM treatment. (**A**) Experimental design for in vivo DXM effects in male rats housed at RT (22 °C) or cold (4 °C). (**B**) Body weight change and (**C**) mean of caloric intake during DXM treatment. Percentage masses of (**D**) RPAT and (**H**) IAT. Representative images from (**E**) RPAT and (**I**) IAT depots. Adipocyte size (diameter) from (**F**) RPAT and (**J**) IAT adipocytes. Representative images from haematoxylin-eosin stained sections from (**G**) RPAT and (**K**) IAT. Magnification ×400. Scale bar: 50 µm. n = 3–4 samples per group. (**L**) Percentage mass and (**M**) representative images from BAT, respectively. In all cases, two-way ANOVA was performed for factors (Cold or DXM) and interaction analysis (Cold × DXM) followed by Tukey’s post-test. Results for factors and interaction are shown in each graph. * *p* < 0.05, *** *p* < 0.001 and **** *p* < 0.0001 vs. CTR-RT. ^++^ *p* < 0.01 vs. CTR-C. n = 10–12 rats per group. Data shown are mean ± SEM.

First, we evaluated the in vivo effects of DXM treatment on several metabolic parameters according to the experimental model shown in Figure 1A. No changes were found in hepatic triglycerides (Tg) and plasma glucose (Glu). However, DXM treatment caused an increase in plasma Tg while Cold exposure exerted the opposite effect (Table 1). As expected, we found that DXM decreased corticosterone (Cort) plasma levels even in cold conditions, where Cort levels were higher than at RT, indicating an inhibition of the hypothalamic-pituitary-adrenal (HPA) axis regardless housing temperatures.

When we analysed the Cold and DXM effects on body weight (BW) gain, we found a significant negative interaction Cold × DXM (Figure 1B), indicating that DXM rats lost less weight than CTR rats when both are exposed to cold, probably due to a browning inhibition. The multiple post-test comparison showed that DXM-RT and DXM-C rats decreased BW to the same extent. Of note, BW change in DXM-RT could be explained, at least in part, by the detrimental effect of DXM in AT, muscle and bone masses, which have been extensively described [28,29].

Regarding caloric intake, DXM did not change it while Cold caused hyperphagia regardless DXM treatment (Figure 1C). On the other hand, both Cold and DXM decreased the Inguinal AT (IAT) and Retroperitoneal (RPAT) percentage masses (Figure 1D,H). When adipocyte size was measured in haematoxilin-eosin (H&E)-stained sections, we observed that RPAT adipocyte size was decreased by Cold, as expected, but also by DXM regardless of housing temperature (Figure 1F,G). This last result could be related to the lipolytic action exerted by DXM, thus decreasing the adipocyte size [28], and/or with the DXM proadipogenic effect, resulting in new and smaller adipocytes [20]. On the other hand, adipocyte size from IAT showed a Cold × DXM negative interaction, indicating that the decrease in DXM-C vs. DXM-RT was lesser than in CTR-C vs. CTR-RT. The post-test showed that CTR-C adipocytes diminished with respect to CTR-RT adipocytes, but that DXM-C showed similar sizes to DXM-RT and to CTR-C (Figure 1J,K). Additionally, BAT mass percentage was calculated and Cold × DXM interaction was found (Figure 1L,M). As expected, the cold increased the mass of BAT, as per thermogenesis activation, while DXM decreased it in DXM-C rats.

### 2.2. DXM Inhibited Browning of IAT and RPAT

**Figure 2:**
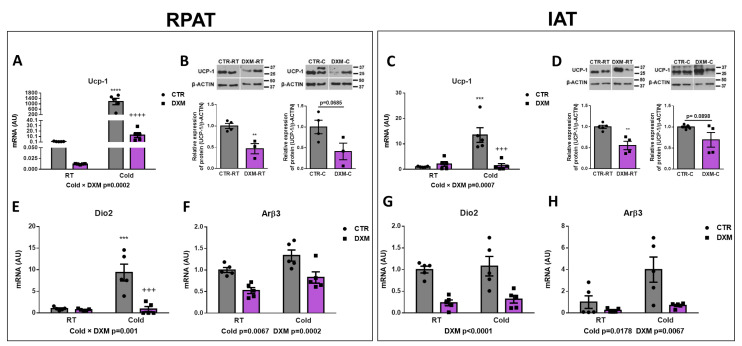
Effect of DXM on cold-induced browning in RPAT and IAT. *Ucp-1* mRNA levels from (**A)** RPAT and (**C**) IAT. UCP1 protein levels from (**B**) RPAT and (**D**) IAT (representative immunoblots and densitometry analysis). β-ACTIN was used as loading control. *Dio2* and *Arβ3* mRNA levels from RPAT (**E**,**F**) and from IAT (**G**,**H**), respectively. Two-way ANOVA was performed for factors (Cold or DXM) and interaction analysis (Cold × DXM) followed by Tukey’s post-test. *** *p* < 0.001 and **** *p* < 0.0001 vs. CTR-RT. ^+++^ *p* < 0.001 and ^++++^ *p* < 0.0001 vs. CTR-C. Results for factors and interaction are shown in each graph. Western blot data were analysed using Student *t*-test. ** *p* < 0.01 vs. CTR-RT. For qPCR quantification, n = 5 samples per group were analysed in duplicate. For Western blot analysis, n = 3–4 samples per group. Data shown are mean ± SEM.

To evaluate the effect of DXM on WAT browning, we analysed different thermogenic markers in RPAT and IAT from basal or cold-stimulated rats treated or not treated with DXM. First, we evaluated the mRNA expression of *Ucp-1* as the main functional marker of thermogenesis. We found that DXM generated a differential response to Cold, decreasing, to a large extent, the mRNA expression of *Ucp-1* in DXM-C mice in both WAT depots (Figure 2A,C). The comparison among groups showed a significant rise of *Ucp-1* expression in CTR-C rats compared to CTR-RT and a decrease in DXM-C compared to CTR-C rats. Furthermore, we observed that the response to cold stimulation was several thousand-fold higher in RPAT than in IAT from CTR-C rats, suggesting that RPAT is thermogenically more active than IAT. UCP-1 protein levels were also evaluated, and showed that UCP-1 levels decreased in RPAT and IAT depots from DXM treated rats in basal conditions (DXM-RT vs. CTR-RT) and showed a strong tendency to low values in stimulated conditions (DXM-C vs. CTR-C, Figure 2B,D). Additionally, the expression of converting T4 to T3 enzyme *Dio2* was quantified. We found that in RPAT, DXM exerted a more marked decreased in mRNA expression in Cold conditions (Cold × DXM interaction), while in IAT caused a proportional inhibition, regardless of housing temperature (Figure 2E,G). Group to group comparison in RPAT showed an increase in *Dio2* mRNA levels in CTR-C vs. CTR-RT rats and a decrease in DXM-C vs. CTR-C rats. Finally, we found that DXM decreased *Arβ3* gene expression at both RT and Cold conditions, while Cold increased it regardless of basal or DXM treatment in both AT depots (Figure 2F,H). Overall, these results indicate that DXM may be exerting an inhibitory effect in RPAT and IAT browning.

### 2.3. DXM Decreased Mitochondrial Content in RPAT and IAT

**Figure 3:**
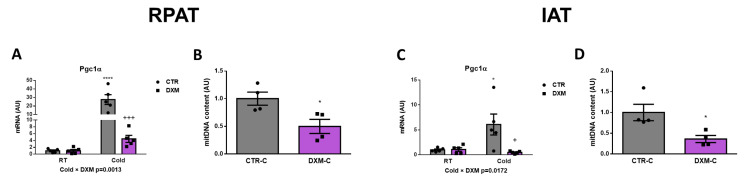
Mitochondrial content in RPAT and IAT from DXM-treated rats. mRNA levels of *Pgc1α* in (**A**) RPAT and (**C**) IAT. DNA mitochondrial content from (**B**) RPAT and (**D**) IAT. Two-way ANOVA was performed for factors (Cold or DXM) and interaction analysis (Cold × DXM) followed by Tukey’s post-test. Results for factors and interaction are shown in each graph. Mitochondrial content was analysed using Student *t*-test. * *p* < 0.05 and **** *p* < 0.0001 vs. CTR-RT. ^+^ *p* < 0.05 and ^+++^ *p* < 0.001 vs. CTR-C. For qPCR quantification, n = 5 samples per group were analysed in duplicate. For mitochondrial content, n = 4 samples per group were analysed in duplicate. Data shown are mean ± SEM.

We wanted to know if DXM affected the mitochondrial content in RPAT and IAT. For this purpose, we evaluated the gene expression of the mitochondrial biogenesis marker *Pgc1α* in both tissues. We found a reduction in *Pgc1α* expression in DXM-C rats from both AT depots (Figure 3A,C). The comparison of *Pgc1α* expression among groups in RPAT and IAT showed similar results: an increase in CTR-C rats and a marked fall in DXM-C rats vs. CTR-C. To further characterize the DXM effect on mitochondria number, we measured mitochondrial DNA content in cold-stimulated conditions, where deeper DXM effects were observed. We found a significant drop in mitochondrial DNA content in both depots (Figure 3B,D). These results suggest that DXM could affect the mitochondrial content, which could contribute to DXM browning inhibition.

### 2.4. Effect of DXM on Gene Expression from Stromal Vascular Fraction (SVF) Cells

**Figure 4:**
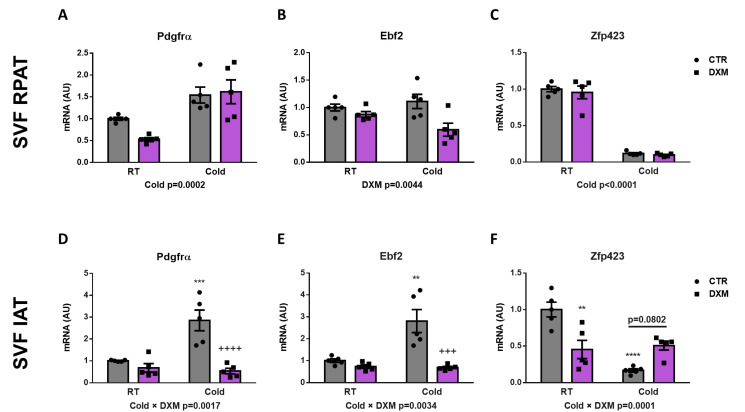
Gene expression of beige precursor markers in SFV cells treated with DXM. mRNA levels of *Pdgfrα*, *Ebf2* and *Zpf423* in SVF cells from (**A**–**C**) RPAT and (**D**–**F**) IAT. Two-way ANOVA was performed for factors (Cold or DXM) and interaction analysis (Cold × DXM) followed by Tukey’s post-test. Results for factors and interaction are shown in each graph. ** *p* < 0.01, *** *p* < 0.001 and **** *p* < 0.0001 vs. CTR-RT. ^+++^ *p* < 0.001 and ^++++^ *p* < 0.0001 vs. CTR-C. n = 5 samples per group were analysed in duplicate. Data shown are mean ± SEM.

To this point, we have evaluated the DXM effect on whole AT. For this approach, we characterised the DXM actions on APCs contained in SVF from both depots, and consequently the generation of new beige adipocytes. To do this, we isolated the SVF from RPAT and IAT and then quantified the gene expression of several beige precursor markers. First, we only observed an increase of *Pdgfrα* expression in SVF cells from RPAT from Cold-exposed rats, independent of DXM treatment (Figure 4A). In IAT SVF cells, we found that *Pdgfrα* expression responded differently to DXM regarding temperature (Cold × DXM interaction), showing a higher suppressive effect of DXM in cold conditions. The results of the post-test analysis indicated increased mRNA levels of *Pdgfrα* in CTR-C rats and decreased in DXM-C animals vs. CTR-C (Figure 4D). Thereafter, we determined the expression of *Ebf2* as the unique selective beige precursor marker described. Interestingly, we found a significant decrease caused by DXM in RPAT cells, independent of temperature condition (Figure 4B). On the other hand, IAT SVF cells showed a differential pattern of *Ebf2* expression, where the suppression caused by DXM was more marked in cells from cold-exposed rats (Figure 4E, Cold × DXM interaction). Multiple comparisons among groups indicated an increased *Ebf2* expression in CTR-C cells and a decrease in DXM-C cells. Finally, we evaluated *Zpf423* expression as a repressor of Ebf2 transcriptional activity, resulting in thermogenic program inhibition. We observed that cold exposure supressed *Zpf423* expression in SVF cells from both fat depots (Figure 4C,F). Regarding Dxm treatment, we did not find changes in SVF cells from RPAT, however we observed, in SVF from IAT, a negative interaction between the variables (Cold × DXM), indicating a smaller decrease in *Zpf423* expression in cold- and DXM-exposed cells. The post-test analyses showed a marked decrease in mRNA levels of *Zpf423* in CTR-C vs. CTR-RT cells and a strong tendency to higher levels in DXM-C vs. CTR-C cells (*p* > 0.0802). These results suggest that the down-regulation of key factors for de novo generation of beige adipocytes and the up-regulation of *Zpf423* could be another mechanism contributing to DXM browning inhibition.

### 2.5. DXM Alters the Expression Pattern of Beige Precursor Cells

**Figure 5:**
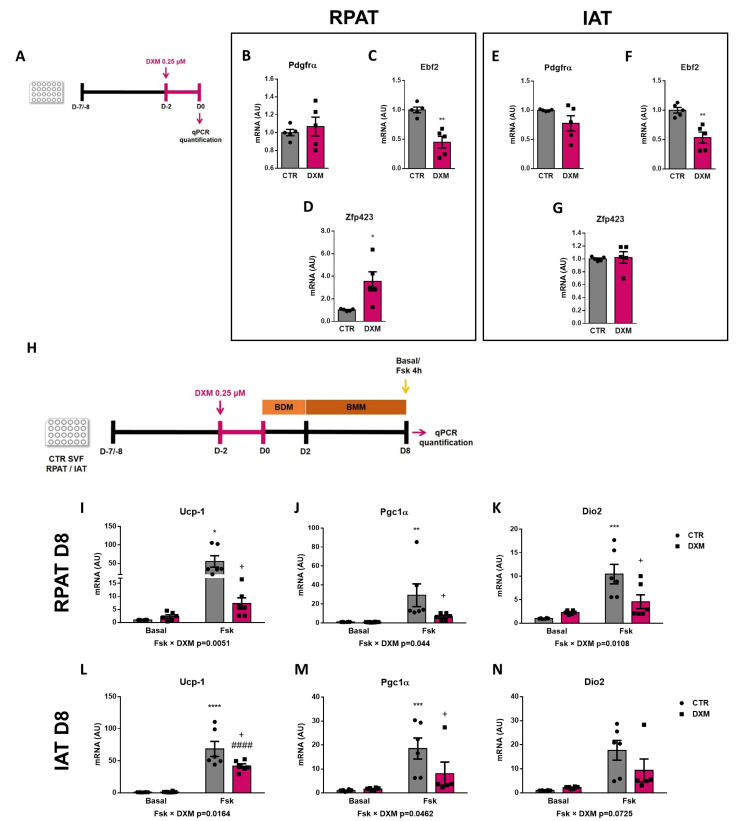
DXM effect on beige precursor expression in cultured APCs. (**A**) Experimental design for in vitro DXM effects on APCs. Briefly, cells were incubated in presence or absence of DXM 0.25 μM (DXM and CTR, respectively) for 48 h. Then, DXM was removed and cells were harvested immediately (D0). BDM: Beige differentiation mix; BMM: Beige Maintenance Medium. mRNA levels of *Pdgfrα*, *Ebf2* and *Zpf423* were quantified in cultured APCs from (**B**–**D**) RPAT and (**E**–**G**) IAT. n = 5 independent experiments were analysed in duplicate. Data was analysed using Student *t*-test. * *p* < 0.05 and ** *p* < 0.01 vs. CTR. (**H**) Additionally, cells were induced to differentiate to beige adipocytes and processed at differentiation day 8 (D8), after stimulation or not with Fsk in the last 4 h (CTR-Fsk and DXM-Fsk, CTR and DXM, respectively). mRNA levels of *Ucp-1*, *Pgc1α* and *Dio2* from (**I**–**K**) RPAT cells and (**L**–**N**) IAT cells. Two-way ANOVA was performed for factors (Fsk or DXM) and interaction analysis (Fsk × DXM) followed by Tukey’s post-test. Results for factors and interaction are shown in each graph. * *p* < 0.05, ** *p* < 0.01, *** *p* < 0.001 and **** *p* < 0.0001 vs. CTR. ^+^ *p* < 0.05 vs. CTR-Fsk. ^####^ *p* < 0.0001 vs. DXM. n = 5–6 independent experiments were analysed in duplicate. Data shown are mean ± SEM.

To better understand the DXM effect on beige precursor cells, we performed an in vitro approach (Figure 5A), wherein RPAT and IAT SVF cells from CTR rats were incubated with or without DXM for 48 h, and then gene expression of beige APC markers were evaluated. We found no changes in *Pdgfrα* gene expression, although *Ebf2* and *Zpf423* expression were decreased and increased, respectively, in DXM treated APCs from RPAT (Figure 5C,D). For IAT, DXM decreased mRNA levels of *Ebf2* without changes in those of *Pdgfrα* and *Zpf423* (Figure 5E–G). These results suggest a direct effect of DXM on the potential of APCs to generate new beige adipocytes. To further test if these changes in APCs’ potential could impact beige adipocytes differentiation, we differentiate DXM-treated APCs with a pro-beige cocktail. We evaluated thermogenic markers on mature adipocytes stimulated or not with forskolin (Fsk) on Day 8 (Figure 5H). First, we observed a differential effect of DXM on the gene expression of *Ucp-1* and *Pgc1α* regarding Fsk stimulation (negative interaction Fsk × DXM), probably indicating that DXM exerted a strong inhibitory effect when adipocytes from both depots are thermogenically active (Figure 5I,J,L,M). When we tested group to group differences, we found, in all cases, an increase in CTR-Fsk vs. CTR adipocytes and a decrease in DXM-Fsk vs. CTR-Fsk adipocytes. Additionally, we quantified *Dio2* expression (Figure 5K,N) and, for RPAT, the results were similar to those found in *Ucp-1* and *Pgc1α*, while in IAT we found a clear tendency to show a negative interaction Fsk × DXM (*p* = 0.0725). Overall, these results robustly suggest that DXM could be reprogramming APCs affecting the generation of beige adipocytes with low thermogenic activity.

### 2.6. DXM Decreased Thermogenic Capacity on Mature Beige Adipocytes

**Figure 6:**
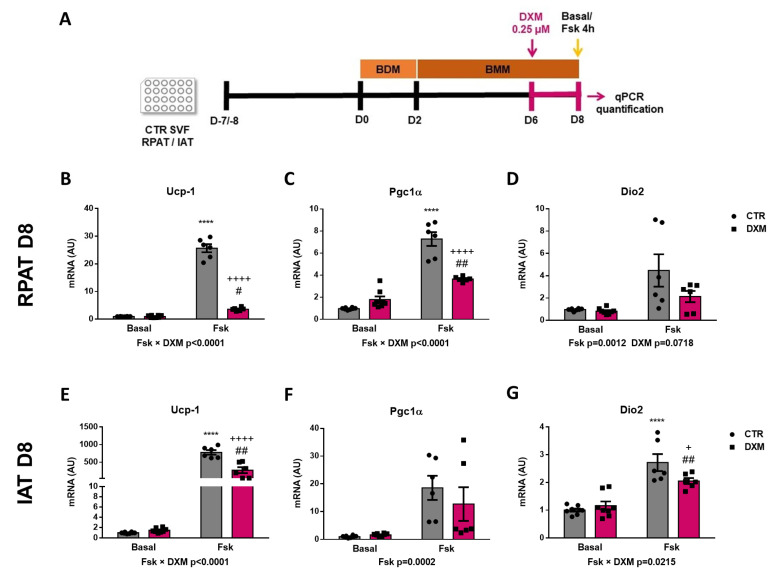
DXM effect on thermogenic program in differentiated beige adipocytes. (**A**) Experimental design for in vitro DXM effects on beige mature adipocytes. Briefly, cells were incubated or not with 0.25 µM DXM the last 48 h of culture and stimulated or not with Fsk the last 4 h (CTR-Fsk and DXM-Fsk, CTR and DXM, respectively). mRNA levels of *Ucp-1*, *Pgc1α* and *Dio2* from (**B**–**D**) RPAT cells and (**E**–**G**) IAT cells. Two-way ANOVA was performed for factors (Fsk or DXM) and interaction analysis (Fsk × DXM) followed by Tukey’s post-test. Results for factors and interaction are shown in each graph. **** *p* < 0.0001 vs. CTR. ^+^ *p* < 0.05 and ^++++^ *p* < 0.0001 vs. CTR-Fsk. ^#^ *p* < 0.05 and ^##^ *p* < 0.01 vs. DXM. n = 6 independent experiments were analysed in duplicate. Data shown are mean ± SEM.

To know if DXM affects the in vitro thermogenic capacity of mature adipocytes, differentiated adipocytes from RPAT and IAT were incubated with or without DXM during the final 48 h in culture. Mature adipocytes were stimulated or not with Fsk and thermogenic markers were evaluated, as outlined in Figure 6A. First, we observed that DXM caused a more marked inhibition in *Ucp-1* expression in adipocytes from both AT depots after thermogenesis stimulation (negative interaction between Fsk and DXM, Figure 6B,E). When compared between different groups, we found an increase in CTR-Fsk vs. CTR adipocytes and a decrease in DXM-Fsk vs. CTR-Fsk adipocytes, but higher levels in DXM-Fsk compared to its basal counterpart. Regarding *Pgc1α*, similar results were found in RPAT as those found in *Ucp-1* expression (Figure 6C); however, in IAT no effects of DXM were noticed (Figure 6F). Moreover, we quantified *Dio2* expression, which showed a similar outcome to that *Ucp-1* for IAT adipocytes, but a tendency to low levels in DXM treated RPAT adipocytes (Figure 6D,G). All these results suggest that DXM also may affect the thermogenic capacity of mature adipocytes when they are treated in a late differentiation stage.

### 2.7. DXM Impacts OCR Profile in 3T3-L1 Adipocytes

**Figure 7:**
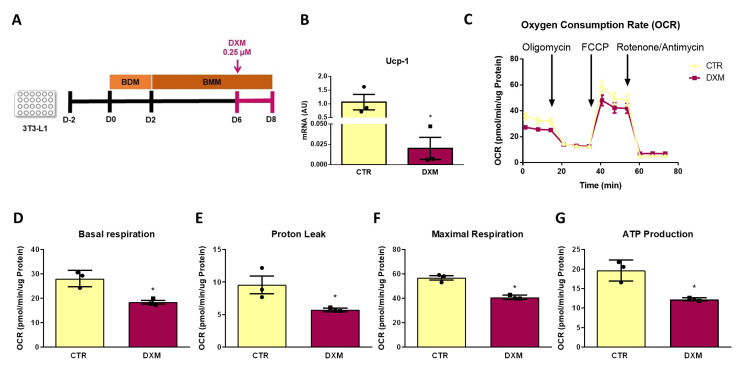
DXM effect on mitochondrial respiration in 3T3-L1 beige adipocytes. (**A**) Experimental design for in vitro DXM effects on 3T3-L1 beige adipocytes. Briefly, cells were incubated or not with 0.25 µM DXM the last 48 h of culture (CTR and DXM, respectively). (**B**) mRNA level of *Ucp-1*. (**C**) Oxygen consumption rate (OCR) profile of CTR and DXM 3T3-L1 beige adipocytes. (**D**) Basal respiration, (**E**) proton leak, (**F**) maximal respiration and (**G**) ATP production parameters from CTR and DXM 3T3-L1 adipocytes. Student *t*-test was used for statistical analysis. * *p* < 0.05 vs. CTR. n = 3 independent experiments in duplicate. Data shown are mean ± SEM.

Finally, we further explored if DXM affects mitochondrial function from beige adipocytes by determining the mitochondrial respiratory capacity. To this end, we measured Oxygen Consumption Rate (OCR) in 3T3-L1 differentiated adipocytes that were treated or not treated with DXM during the final 48 h of culture, as shown in Figure 7A. Prior to the respiration measurement, we quantified *Ucp-1* expression to corroborate the same effect of DXM found in primary adipocytes. As shown in Figure 7B, DXM effectively supressed *Ucp-1* gene expression in 3T3-L1 adipocytes. We then evaluated the OCR profile and the respiration parameters obtained from it (Figure 7C–G, respectively). We noticed that DXM decreased all respiration parameters, including the proton leak, which is directly related to the presence of UCP1 in the inner mitochondrial membrane (Figure 7E). These findings confirmed that DXM affects the thermogenic capacity of mature beige adipocytes.

## 3. Discussion

DXM is a synthetic GC that binds with higher affinity to GCs receptors and thus has more potent GC actions. DXM is widely used in clinical treatments as an anti-inflammatory agent, however its chronic usage is related to undesirable side effects, most of them associated to AT dysfunction [30,31], such as AT abdominal hypertrophy and insulin resistance, among others. On the other hand, WAT browning activation has emerged as a therapeutic target due to its potential role against obesity. It has been stated that AT specific transgenic mice overexpressing UCP-1 showed decreased body weight and low fat mass in both male and female mice [32]. In addition, transgenic mice overexpressing Prdm16 in WAT, thus with increased beige fat function, gained less body weight during high-fat diet (HFD) and showed less fat mass [7]. However, the relationship between GCs actions on non-shivering thermogenesis remains largely unexplored, and for this reason we aimed to elucidate the DXM effects on WAT thermogenesis and on the activation/generation of beige adipocytes.

It is important to note that most of the existing studies on GCs and DXM actions in thermogenesis has been in regards to BAT. In rodents under normal diets or HFD, treatment with DXM decreases BAT *Ucp-1* mRNA levels and energy expenditure [22]. Similar results were found in mice and rats treated with corticosterone (subcutaneous pellet), where mRNA expression and UCP-1 protein content of BAT [33] and GDP binding mitochondria were reduced [34]. However, Luijten et al. found no changes in total UCP-1 protein levels in BAT from GCs treated mice (through the drinking water) and housed at 21 °C, while at thermoneutrality a suppressive effect was noticed [35]. In humans, acute treatment with prednisolone increases energy expenditure and supraclavicular temperature under moderate cold exposure (16–17 °C), while chronic oral glucocorticoid treatment suppresses BAT activation [36]. These contradictory results may be due to different treatment approaches (duration: acute vs. chronic treatments, housing temperature) or even different responses among species (rodents vs. humans), as previously reported [36]. Here we found that rats exposed to cold conditions and treated with DXM showed lower BAT mass than CTR-C rats, which may be in line with a reduced BAT activity; however, we did not evaluate BAT activation or UCP-1 content because it was beyond of our study aim.

As mentioned before, there is little information about browning of WAT and GCs effects. In a previous study, we found that hypercorticosteronemic rats expressed less GR in WAT and increased thermogenic markers, which was reversed when GR expression was normalized after adrenalectomy [37]. Some authors found low expression of *Ucp-1* in subcutaneous and visceral WAT from mice treated with DXM, an effect that could be mediated by miR-27b, an upstream regulator of Prdm16 in WAT [26]. It is important to highlight that most studies were conducted at RT or thermoneutrality, where the number and activation of beige adipocyte are scarce. In this work, we evaluated the effect of DXM treatment at basal (RT) but also in cold stimulated thermogenesis, where WAT browning is physiologically relevant. We found that DXM inhibited WAT browning from both depots even more in cold stimulated than in RT conditions. Our results are in line with a previous report by van den Beukel JC. et al. that describes a reduction of *Ucp-1* gene expression and protein staining in inguinal WAT from corticosterone treated mice at both 23 °C and 4 °C [38]. Additionally, we described that DXM reduced the mitochondrial content and decreased the expression of the mitochondrial biogenesis marker *Pgc1α* (RPAT) in cold stimulated conditions. It is worth noting that our results support the idea that, in rats, RPAT has higher thermogenic response than IAT, in contrast to that found in mice, where subcutaneous WAT is highly prone to browning. Previously, we reported similar results in WAT browning from rats [39]. Overall, these results suggest an inhibitory effect of DXM on WAT browning, especially after cold stimulation in an abdominal WAT depot.

Beige adipocytes can be generated by two different mechanisms: the differentiation of adipocyte precursor cells to beige adipocytes or transdifferentiation of white-to-beige adipocytes [10]. It has been described that the contribution of each mechanism to WAT browning depends on the previous stimuli: initially upon the first cold exposure, beige adipocytes originate in a greater proportion from de novo beige adipogenesis, then upon subsequent stimuli arise from differentiated unilocular adipocytes (white or dormant beige adipocytes) [40]. Little is known regarding DXM’s actions on the mechanism of beige adipocytes generation. Indeed, to our knowledge, this is the first study addressing DXM direct actions on beige progenitor cells. We found that DXM decreased the expression of *Ebf2* in precursors from RPAT and IAT, which was proposed as a selective marker of beige precursors [12]. On the other hand, ZFP423 defines the commitment of white APCs and also maintains mature white adipocytes features through suppression of thermogenic program [14,41]. Specifically, interaction between ZFP423 and EBF2 prevent the PPARγ2 thermogenic program activation, thus favoring white adipocyte phenotype; meanwhile, the lack of ZFP423 induces the activation of thermogenic genes by PPARγ2 [15]. We found an increase in *Zpf423* mRNA levels in RPAT DXM-treated cells (in vitro approach) and an attenuated *Zpf423* expression drop in SVF cells from IAT after cold exposure (in vivo approach). Our results concur with previous reports from our group and another study that showed a positive effect of DXM on *Zpf423* expression [41,42]. No differences were found in the bipotential white-beige marker *Pdgfrα*; this may be related to the fact that DXM activates white progenitors [42] but seems to repress the beige precursors. In addition, we evaluated how the imprinting of DXM in beige precursors could affect the later differentiation. This approach resulted in the generation of adipocytes with low gene expression of *Ucp-1*, *Pgc1α* and *Dio2* in both WAT depots, mainly in Fsk-stimulated conditions. Taken together, these results suggest that DXM could be affecting beige adipogenic potential and differentiation capacity, especially when the thermogenic process is activated.

In a previous report, Lv YF. et al. showed an inhibition of the thermogenic program in brown and IAT beige adipocytes differentiated in the presence of DXM, probably mediated by miR-19b, the upstream regulator of ARβ1 [25]. Here, we studied the effect of DXM on terminal differentiated beige adipocytes, allowing us to evaluate whether DXM affects mature beige functionality in basal and thermogenic conditions. We found an inhibition of the thermogenic program, which was even more evident in Fsk-stimulated adipocytes, mainly in RPAT DXM-treated cells. Finally, measurement of the oxygen consumption rate in 3T3-L1 differentiated to beige adipocytes confirmed the inhibitory effect of DXM in mitochondrial respiration, decreasing all parameters related to mitochondrial bioenergetics. The proton leak, which is related to UCP-1 content in the inner mitochondrial membrane, and the maximal respiration, which reflects the mitochondrial number and/or the electron transporter proteins content, were both decreased in DXM-treated cells. Our data are in agreement with previous findings showing a reduced oxygen consumption rate in murine brown and beige adipocytes treated with DXM during adipogenic induction [25].

In conclusion, we demonstrated that DXM inhibited the thermogenic program in WAT, especially in stimulated conditions. This effect could be related to a decreased mitochondrial content. Additionally, in vitro data indicated that DXM decreased both beige precursor marker expression in APCs and mature beige adipocyte activation, indicating that DXM may inhibit WAT browning by affecting both cellular targets. However, as a limitation of our study, we only focused on the study of DXM effects in male rats. Taking into account that GCs, and specially DXM actions, are sexually dimorphic [43,44,45] and that the thermogenic activities of BAT and WAT are also gender dependent [46,47], it would be interesting to evaluate and compare the DXM effects in the thermogenic program of female rats.

As mentioned above, GCs are still used for treatment of diverse pathologies, mainly due to their anti-inflammatory actions [48]. Unfortunately, prolonged treatments with GCs, particularly with DXM, trigger different undesirable effects, mainly associated with hypertrophic WAT mass expansion and consequently an altered adipokine secretion pattern [49]. These AT dysfunctions favor the development of cardiovascular diseases, dyslipidemia and insulin resistance among other effects, as seen in Cushing’s Syndrome patients [50]. In this context, our findings could demonstrate a new deleterious effect of chronic exposure to GCs, since the inhibition of WAT browning might contribute to reduced energy expenditure, thus favoring the hypertrophic expansion of WAT and therefore aggravating several metabolic alterations.

## 4. Materials and Methods

### 4.1. Animals and Experimental Design

Male Sprague–Dawley rats, 60-days old and weighting 200–350 g, were used in the experiments. Animals were housed one per cage in a controlled environment (23 ± 1 °C, 12 h light-dark cycle, lights on at 7 A.M.). One week before the experiments began, they were handled over 3 days to habituate them and minimize stress responses during experimental procedures. Then, rats were divided into four groups: control (CTR-RT) and DXM injected daily (sc 0.03 mg/Kg/d for 7 days, DXM-RT) and housed at room temperature, and CTR and DXM housed under cold stimulus (4 °C for 7 days, CTR-C and DXM-C, respectively). Food intake and body weight were recorded every day. On the experimental day, rats in non-fasting conditions (between 08:00 and 09:00 h) were euthanized and trunk blood was collected; plasma samples were then frozen (−20 °C) until metabolite measurements were conducted. RPAT, IAT and BAT depots were dissected, weighed and processed for several determinations. RPAT and IAT were kept in Dulbecco’s Modified Eagle’s Medium-Low Glucose (1 g/L) (DMEM) for SVF cells isolation and further procedures (RT-qPCR and Western Blot assays). Additionally, control male adult S-D rats (60-day old) were euthanized under non-fasting conditions (between 08:00 and 09:00 h). RPAT and IAT pads were aseptically dissected and placed into sterile conic tubes containing 10 mL of sterile DMEM for further SVF cells isolation. Isolated SVF cells were cultured and used for in vitro experiments. All animals were euthanized according to protocols for animal use, in agreement with National Institutes of Health guide for the care and use of laboratory animals. All experiments were approved by our Institutional Animal Care Committee (Approval Code 050916).

### 4.2. Metabolic Parameter Measurements

Circulating glucose (Glu) and triglycerides (Tg) levels were measured using commercial kits (WienerLab., Rosario, Argentina). Plasma corticosterone (Cort) was measured by specific radioimmunoassay (RIA) previously developed in our laboratory [51].

### 4.3. Liver Lipid Content

Fifty mg of the liver was homogenized in a 5% solution of 500 μL Triton X-100 in PBS. The homogenate was incubated at 80–100 °C for 5 min and centrifuged at 10,000× *g* for 10 min. Tg was measured in the supernatant using a commercial kit (WienerLab., Rosario, Argentina).

### 4.4. Histological Analysis

RPAT and IAT samples from four groups of rats were fixed in 4% formaldehyde in PBS for 24–48 h, dehydrated in several ethanol gradients, cleared in xylene and embedded in paraffin. Tissue sections (5 µm) were stained with hematoxylin-eosin [52]. Quantitative morphometric analysis was performed using a RGB CCD Sony camera and the automated Open Sources Software Adiposoft as a plug-in for Fiji software 1.54f [53] (magnification ×400). For each tissue sample, seven sections and three levels were selected (n = 3 animals per group). Systematic random sampling was used to select fields for each section, and 3000 cells per group were examined. Adipocyte diameter was measured [54].

### 4.5. RPAT and IAT Stromal Vascular Fraction Cell Isolation

Fresh RPAT and IAT pads were dissected, weighed and digested with collagenase Type I, as previously reported [55]. Briefly, fat tissues were minced and digested using 1 mg/mL collagenase solution in DMEM (at 37 °C, for 1 h). After centrifugation (1000 rpm, during 15 min), floating mature adipocytes were discarded. SVF pellets were filtered (in a 50 μm mesh nylon cloth) and washed with DMEM (×3). SVF cells were then resuspended in DMEM supplemented with 10% (*v*/*v*) fetal bovine serum (FBS), HEPES (20 nM), 100 IU/mL penicillin and 100 μg/mL streptomycin (basal medium) and reserved for further measurement. SVF cells from CTR rats were seeded (2 × 10^4^ cells/cm^2^) in 24-well plates and cultured up to confluence (5 days) in DMEM supplemented with 10% (*v*/*v*) FBS and antibiotics at 37 °C in a 5% CO_2_ atmosphere [55]. Then, cells were used for two experimental designs. First, to evaluate the effects of DXM on APCs and posterior beige adipocyte differentiation, cells were incubated in presence or absence of DXM 0.25 μM (DXM and CTR, respectively) for 48 h. Then, DXM was removed and cells were harvested immediately (D0) (Figure 5A) or were induced to differentiate by the addition of beige differentiation mix (BDM) containing 5 µg/mL insulin, 2.5 μM DXM, 0.5 mM 3-isobutyl-L-methylxanthine (IBMX), 1 μM rosiglitazone and 0.1 nM triiodothyronine (T3) for 48 h. Afterwards, BDM was removed and replaced with fresh medium containing 5 µg/mL insulin and T3 (Beige Maintenance Medium, BMM). On day 8 after differentiation (D8), a subset of CTR and DXM cells were incubated in basal condition or with Fsk (10 µM) for 4 h (CTR-Fsk and DXM-Fsk, respectively) (Figure 5H). Cells were then processed for qPCR quantification of several thermogenic markers. Additionally, and following a similar protocol, to evaluate DXM effect on transdifferentiation or thermogenesis of mature beige adipocytes, cells were incubated or not with 0.25 µM DXM the last 48 h of culture (Figure 6A).

### 4.6. RNA Isolation and Quantitative Real-Time PCR (qPCR)

Total RNA was isolated from tissues and cells by the Trizol extraction method (Invitrogen, Life Tech., Waltham, MA, USA). Total RNA was reverse-transcripted using random primers (250 ng) and RevertAid Reverse Transcriptase (200 U/μL). One μL of cDNA was amplified with HOT FIRE Pol EvaGreen qPCR Mix Plus (Solis BioDyne, Tartu, Estonia) containing 0.5 μM of each specific primer using a Rotor-Gene-Q Real Time PCR cycler (Qiagen, Hilden, Germany). PCR efficiency was near 1. Expression levels were analysed for β-actin (*Actβ*, reporter gene), *Arβ3*, *Dio2*, *Ebf2*, *Pgc1α*, *Pdgfrα*, *Zfp423* and *Ucp-1* (designed primers are shown in alphabetical order in Table 2). Relative changes in the expression level of each specific gene (ΔΔCt) were calculated by the ΔCt method.

### 4.7. Western Blotting Assays

Tissue samples were homogenized by sonication in RIPA buffer at 4 °C supplemented with protease and phosphatase inhibitors cocktail. Tissues extracts were centrifuged at 12,000 rpm at 4 °C for 20 min to remove insoluble material. After quantification, 20 µg of total protein was subjected to polyacrylamide gel electrophoresis (SDS PAGE 12%) and transferred to a polyvinylidene fluoride membranes (PVDF). Then, membranes were blocked 1 h in 5% BSA-TBST, 20 mM Tris-base, pH 7.6, 137 mM NaCl and 0.1% Tween 20 and incubated with the respective primary antibody β-ACTIN (bs-0061R; lot#AH01032107, Bioss antibodies, Woburn, MA, USA, 1/4000) and anti-UCP-1 (ab-10983, lot#GR214286-5; Abcam, Cambridge, UK, 1/3500) overnight at 4 °C. After washing, membranes were incubated with Horseradish Peroxidase conjugated anti-rabbit (sc-2004, lot#22905; Santa Cruz, Dallas, TX, USA, 1/10,000) as secondary antibody for 2 h at room temperature with constant shaking. The protein signals were detected using ECL and autoradiography films (Carestream Medical X-ray Green/MXG film). Images were analysed with Fiji software 1.54f.

### 4.8. Mitochondrial DNA Quantification

RPAT and IAT pads from CTR-C and DXM-C were homogenized and sonicated with lysis buffer (40 mM EDTA, 50 mM NaCl, 100 mM Tris, 0.2% SDS) in PBS (2:1 at cold temperature, proteins were precipitated with 5.4 M NaCl, centrifuged at 8000 rpm for 20 min and soluble DNA was precipitated with cold isopropyl alcohol. Finally, DNA was dissolved in TE buffer and quantified by spectrophotometer at 260 nm. For PCR reactions, 5 ng was used for RNAt-Leu (mitochondrial gene, se: GGTGGCAGAGCCAGGTAAT and as: AGGATTTGAACCTCTGGGAAC primers) and β2-microglobulin (nuclear gene, se: CCCTATAGTGTGCGGTCGTT and as: GACGAGCATGCACTCCACTA primers) determinations. Relative changes in the DNA mitochondrial content were calculated by the ΔΔCt method.

### 4.9. Gene Expression Analysed in 3T3-L1 Cells

Cells line 3T3-L1 were seeded at 1000 cells/well in a culture plate. Then, cells were differentiated and treated or not with DXM 0.25 μM (DXM and CTR, respectively) the last 48 h of culture (Figure 6A). On the experimental day, cells were harvested and processed for RT-qPCR real time, as explained before. Expression levels were analysed for Ucp1 (se: AAACTGTACAGCGGTCTGCC and as: AAGCCGGCTGAGATCTTGTT) and *Actβ* as reporter gene (se: TGGCGCTTTTGACTCAGGAT and as: GGGATGTTTGCTCCAACCAA).

### 4.10. Oxygen Consumption Rate (OCR) Determination

Cells line 3T3-L1 were seeded at 1000 cells/well in a XFp microplate. Then, cells were differentiated and treated or not with DXM 0.25 μM the last 48 h of culture (Figure 6A). On the experimental day, cells were washed and stabilised, and oxygen consumption rates (OCR) were performed using the Seahorse XFp analyser (Agilent, Santa Clara, CA, USA), following the manufacturer’s instructions. First, basal respiration was measured, and then cells were incubated with oligomycin (2 μM, an ATP synthase inhibitor), FCCP (1.5 μM, an uncoupler of oxidative phosphorylation) and rotenone/antimycin A (2 μM/2 μM, complex I/III inhibitors). All experiments were performed at 37 °C. For respiration parameters [56], non-mitochondrial OCR was obtained after the addition of rotenone/antimycin and subtracted from baseline OCR (basal respiration) and OCR after the addition of FCCP (maximum respiration). Spare respiratory capacity was calculated as maximum respiration minus basal respiration.

### 4.11. Statistical Analysis

Results were expressed as mean values ± S.E.M. Two-way ANOVA was performed for factorial analysis, including two factors and two levels each: DXM and Cold effects (in vivo experiments) and DXM and Fsk effects (in vitro experiments) and the interaction between the two factors (DXM × Cold or DXM × Fsk, respectively). Only when interaction was statistically significant, Tukey’s post-hoc test was performed for comparisons of between groups [57]. Statistical differences between two groups were evaluated using Student’s *t* test. In all cases, *p* values lower than 0.05 were considered statistically significant. All statistical tests were performed using GraphPad Prism version 6.00 for Windows (GraphPad Software, San Diego, CA, USA).

## Figures and Tables

**Table 1:** Metabolic parameters from CTR-RT, DXM-RT, CTR-C and DXM-C rats.

Variable	CTR-RT	DXM-RT	CTR-C	DXM-C	2-Way ANOVA		
					Cold	DXM	ColdxDXM
Plasma Glu (g/L)	1.27 ± 0.05	1.25 ± 0.02	1.33 ± 0.03	1.25 ± 0.04	ns	ns	ns
Plasma Tg (g/L)	1.13 ± 0.10	1.72 ± 0.19	0.28 ± 0.03	0.54 ± 0.09	*p* < 0.0001	*p* < 0.01	ns
Liver Tg (mg/g Liver)	5.83 ± 0.96	5.77 ± 0.84	4.22 ± 0.23	5.09 ± 0.19	ns	ns	ns
Cort (ng/dL)	4.52 ± 0.89	2.62 ± 0.37	7.79 ± 2.02	4.22 ± 0.93	*p* < 0.05	*p* < 0.05	ns

Two-way ANOVA analysis. Values are mean ± SEM. n = 10–12 per group. Plasma determinations were measured in duplicate. Experimental groups: CTR and DXM rats housed at RT (CTR-RT and DXM-RT, respectively) or cold (CTR-C and DXM-C, respectively). ns: not significant.

**Table 2:** Rat-specific primers for qPCR listed in alphabetical order.

	Primers (5′-3′)	GBAN	bp
*Actβ*	se, AGCCATGTACGTAGCCATCC	NM_031144	200
	as, ACCCTCATAGATGGGCACAG		
*Arβ3*	se, AGTGGGACTCCTCGTAATGC	NM_013108	110
	as, TTACACAGAGCACGTCCACT		
*Dio2*	se, CTGGCGCTCTATGACTCGG	NM_031720	194
	as, ACGTGCACCACACTGGAAT		
*Ebf2*	se, TCTTATCCTACATCCCACACCC	NM_001108383.1	113
	as, TGAGTCTGGTTTCTGTGGTGG		
*Pdgfrα*	se, AGGCTTGGGGCTCACTTTTT	NM_012802	153
	as, AAGAGCTGGCAGACGATGAG		
*Pgc1α*	se, AAAAGCTTGACTGGCGTCAT	NM_031347	199
	as, ACACCACTTCAATCCACCCAG		
*Ucp-1*	se, CCGAAACTGTACAGCGGTCT	NM_012682	155
	as, GTCATCAAGCCAGCCGAGAT		
*Zfp423*	se, CCGCGATCGGTGAAAGTTG	NM_001393718.1	121
	as, CACGGCTGGTTTTCCGATCA		

se: sense; as: anti-sense; GBAN: GenBank Accession Number; amplicon length in bp.

## Data Availability

Data is contained within the article.
